# Resisting Moralisation in Health Promotion

**DOI:** 10.1007/s10677-018-9941-3

**Published:** 2018-11-08

**Authors:** Rebecca C. H. Brown

**Affiliations:** Oxford Uehiro Centre for Practical Ethics, University of Oxford, Suite 8 Littlegate House, 16-17 St Ebbe’s Street, Oxford, OX1 1PT UK

**Keywords:** Public health ethics, Moralisation, Moralism, Health promotion, Health policy, Responsibility

## Abstract

Health promotion efforts are commonly directed towards encouraging people to discard ‘unhealthy’ and adopt ‘healthy’ behaviours in order to tackle chronic disease. Typical targets for behaviour change interventions include diet, physical activity, smoking and alcohol consumption, sometimes described as ‘lifestyle behaviours.’ In this paper, I discuss how efforts to raise awareness of the impact of lifestyles on health, in seeking to communicate the (perceived) need for people to change their behaviour, can contribute to a climate of ‘healthism’ and promote the moralisation of people’s lifestyles. I begin by summarising recent trends in health promotion and introducing the notion of healthism, as described by Robert Crawford in the 1980s. One aspect of healthism is moralisation, which I outline (alongside the related term moralism) and suggest is facilitated by efforts to promote health via information provision and educational strategies. I propose that perceived responsibility plays a role in mediating the tendency to moralise about health and behaviour. Since I argue that states ought to avoid direct and indirect moralisation of people’s health-related behaviour, this suggests states must be cautious with regard to the use of responsibility-indicating interventions (including informational and educational campaigns) to promote health.

## Background: Public Health and the ‘Behavioural Turn’ in Health Promotion

A great success of modern healthcare has been the vast improvements in human health and well-being achieved through preventative public health strategies. Historically, big gains in public health have come about through improvements to living conditions and sanitation, alongside population vaccination programs (Mold and Berridge 2011). Although infectious disease outbreaks still persist, resulting in numerous premature deaths (particularly in areas of rapid urbanisation), public health measures, amongst other socio-economic factors, have helped to reduce rates of mortality from infectious disease across the globe (WHO 2008). Life expectancies have increased as a result, and so too has the proportion of morbidity and mortality resulting from chronic disease, now the leading cause of death globally (WHO 2014).

This has led to a shift in the focus of public health. Modern public health is now often directed towards health promotion, and focuses less on prevention and eradication of disease, and more on reducing lifestyle risk factors for chronic disease (Mair 2011; Crawshaw 2013). The four major chronic diseases are heart disease, cancer, lung disease and diabetes. Epidemiologists have identified a number of behavioural risk factors which make it more likely that someone will suffer from one or more of these diseases during their lifetime. These risk factors include poor diet, lack of physical activity, smoking and alcohol consumption (Ezzati and Riboli 2013). In order to reduce and postpone the harmful impact of chronic disease on people’s lives, much health promotion is now focused on encouraging individual behaviour change – i.e. getting people to alter their lifestyles and adopt healthier habits.

Social historians have described this shift as the ‘behavioural turn,’ occurring in the UK in the late twentieth century, and characterised by a move away from a form of public health focused on the provision of social goods and the contributions these can make to health, and instead emphasising individual responsibility for making healthier choices (Crawford 1977; Crawshaw 2013; Mair 2011). This has been mirrored by broader social trends within politically ‘western’ democracies towards incorporating the neoliberal values of individual choice and empowerment into policy, such as the ‘choice agenda’ of the 1997 New Labour government in the UK (Le Grand 2007; Taylor-Gooby 1998; Jordan 2005).

Since the behavioural turn, a significant focus of health promoting behaviour change has focused on ‘empowering healthy choices’ through information provision and educational campaigns (supposedly acting to widen people’s choice set and ability to select according to their preferences). Alternative approaches have also been used. In particular, policy may restrict or remove choice by regulating the sale of products or the permissibility of the behaviour itself. This includes increasingly restrictive regulation on the sale of tobacco and alcohol, the banning of trans fats from processed foods, and the outlawing of smoking in public places. Such policies are often described as coercive since they limit people’s freedom of choice, with the threat of legally enforced punishment for failure to comply.

As well as informational and coercive interventions, more recently, interest has turned to methods of influencing behaviour via unconscious processes. Particularly influential has been the work of Richard Thaler and Cass Sunstein in promoting the idea of the ‘nudge’ (Thaler and Sunstein 2008). Roughly, nudges are interventions which alter, often in subtle ways, the environment in which a behavioural choice is made. In doing so, they shape people’s behaviour, nudging them towards healthier choices. The classic example of a health nudge is using the layout of food items in a cafeteria in order to encourage people to buy healthier options. Supposedly, the prominence and ordering of different foods can affect what people will buy, so alterations to what Thaler and Sunstein call the ‘choice architecture’ (in this case, the layout of the cafeteria) can impact behaviour.

Unsurprisingly, both historical and contemporary ethical criticism of public health interventions have tended to focus on coercive policies, such as mandatory vaccination programs and legal restrictions on the purchase and sale of consumables (Luyten et al. 2011; Breton and Sherlaw 2011). The threat such policies are assumed to pose to freedom makes them an obvious target for critics concerned about inappropriate state intervention. More recently, the proposed use of nudges and other tools for influencing behaviour with limited or no conscious awareness of those being influenced, has stoked fears that they involve illegitimate manipulation of people’s behaviour and, thus, undermine autonomy (Hausman and Welch 2010; Quigley 2014; Wilkinson 2012).

In contrast, public health promotion which has focused on the provision of health related information and the delivery of educational campaigns to raise awareness of behavioural risk factors has typically been seen as less objectionable. These interventions are explicitly presented as efforts to facilitate people in making independent, well-informed choices; to preserve freedom and promote autonomy. As such, these interventions tend to be seen as benign, and attract far less (ethical) criticism.

In this paper, I question whether such approaches to health promotion are as benign as they are often taken to be. I propose that an effect of focusing on behaviour change through information provision and educational campaigns may be to support the moralisation of people’s lifestyles. I argue that such health promotion efforts increase the view that people *can* and *should* adopt healthy behaviours, and in doing so, reinforce (misconceived) beliefs regarding people’s responsibility for their health-related behaviour and subsequent (ill) health, and foster a moralising discourse of health-related behaviours. Further, I suggest that, in general, states should refrain from direct or indirect moralising in relation to health, and that this has implications for the supposedly ‘benign’ use of informational and educational strategies as a way of tackling chronic disease. Whilst I do not discuss them directly, this criticism suggests that if alternative approaches to health promotion (such as regulation and the use of environmental shaping to influence behaviour) avoid contributing to moralisation, there may be ethical reasons for preferring such approaches to health promotion.

## Healthism and the Promotion of Healthy Choices

In pursuing reductions in chronic disease, health promoters have sought to raise awareness of behavioural risk factors, in particular unhealthy diets, lack of physical activity, tobacco smoke and excessive alcohol consumption. Online resources, such as the NHS Choices *Live Well* website, provide tips on weight loss through at-home fitness workouts and healthy recipes, as well as guidelines about alcohol consumption, and information on the harmful effects of smoking (NHS 2016). Interventions at educational establishments and the workplace, informational leaflets, posters, product packaging, and online and television adverts are used by health promoters to convey messages about the harms of unhealthy habits, and to encourage people to make healthy changes to their lifestyles.

The pervasiveness of health messages, touching upon almost all areas of a person’s life, is part of a phenomenon sometimes described as ‘healthism.’ One of the first to use the term healthism was Robert Crawford (1980). In relation to what he describes as the new ‘health consciousness’ and related movements of holistic medicine and self-care in the US during the 1970s, Crawford argues that “The concern with personal health has become a national preoccupation. Ever increasing personal effort, political attention, and consumer dollars are being expended in the name of health.” (Crawford 1980: 365) Healthism includes 1. the tendency to view an increasing number of activities and domains of life in terms of the impact they have on health, and 2. the promotion of health to a ‘super-value.’

This first component of healthism is akin to a kind of medicalisation, whereby domains of life previously not considered relevant to health, or as appropriately falling within the structures of healthcare, are newly seen through a medical lens. This results in the extension of the category of ‘health-related,’ and encourages a disproportionate focus on the health-affecting relevance of particular things (often, whilst neglecting their wider significance).

The second sense of healthism elevates the value of health, often now expanded to include mental and other kinds of well-being, to the status of super-value. Those who fail to value it properly, as demonstrated, for instance, by failing to take steps to preserve their own health, become seen as imprudent at best, reckless and deviant at worst. As Crawford describes:

[H]ealth has become not only a preoccupation; it has also become a pan-value or standard by which an expanding number of behaviors and social phenomena are judged. Less a means toward the achievement of other fundamental values, health takes on the quality of an end in itself. Good living is reduced to a health problem, just as health is expanded to include all that is good in life.

Crawford 1980: 380-381

These two aspects of healthism are mutually reinforcing: the expanding scope of the ‘health-related’ domain encourages the view that health is a (perhaps *the*) dominant value which everyone should pursue, and which determines the way in which we should assess the worth of all other things. Likewise, the emphasis on the universal importance of health pushes us to identify how any and all of our (and others’) activities impact on health.

Whilst Crawford diagnosed healthism in the health consciousness movements of 1970s America, something similar can be seen in relation to the behavioural turn in public health promotion, and the focus of contemporary health promotors on providing information and education to people about the harms of unhealthy lifestyles. The discovery of links between chronic disease and behaviours such as diet, exercise, smoking and drinking, has brought those ‘lifestyle’ behaviours into the health domain. The continued elevation of the value of health and well-being, with the addition of now viewing risk-factors for disease as almost equivalent to disease itself, redescribes ‘healthy’ as not merely(!) freedom from disease, along with complete mental and physical well-being, but now freedom from *risk of future* disease (Crawshaw 2013).

I suggest that this approach to public health promotion will often, directly or indirectly, support moralisation of people’s behaviour. I do not endorse or defend a particular concept of health (or disease) here. Whilst I have no principled objection to a number of plausible approaches to defining health (including, for instance, those emphasising freedom from disease, attainment of well-being, species typically norms and at least some role for social construction),^1^ the adoption of a particular concept – either explicitly or implicitly through health-related practices – could play a role in the processes of healthism and moralisation I describe. Roughly, broader concepts of health and/or those specifying health as a priority over other goods will align with healthism and foster moralisation to a greater extent. From a pragmatic perspective, therefore, it may be wise to narrow the scope of health to some extent, and to acknowledge plural (non-health) values. In the next section, I outline what moralisation involves, before going on to indicate how it is fostered by informational/educational strategies to tackle chronic disease.

## Moralisation

Moralisation and its close relations – moralism, moralist, moralise, etc. – are applied in different ways in various academic discussions and everyday contexts. In this section I will provide a rough definition of what I take moralisation (and its connected terms) to mean, and discuss their relevance to state health promotion.

Mostly used within the social sciences, moralisation has been described as “the acquisition of moral qualities by objects and activities that were previously morally neutral.” (Rozin 1997: 380) Thus, the process of moralisation brings within the moral domain activities that previously fell outside of it. This primarily describes a social process, by which activities come to acquire moral status within a particular social/cultural context at a particular time. Psychologists have also sought to describe moralisation at the individual level: Paul Rozin describes how individuals may come to assign moral status to activities either through cognitive, reason-based practices, or through affective, emotional associations (Rozin 1997: 385–386). Once again, this refers to the same process, enacted at the individual rather than group level.

It should be noted that *any* activity can be argued to have potential moral relevance, given particular contextual features. For instance, John’s decision about what brand of coffee to purchase may seem morally inert, but if we stipulate that one brand routinely exploits its workers and fails to pay them a fair wage, whilst the alternative is a conscientious employer, then his decision may take on additional moral significance. It is therefore unclear that any activity is ever truly ‘outside’ the moral domain, although it may be less commonly viewed as morally salient, and less subject to moral criticism or praise. Changes to contextual factors and the action of social norms will thus dictate whether or not something is considered to fall within the ‘moral domain.’

As used within the social sciences, discussions of moralisation typically resist making any normative judgements about the process of moralisation itself. In common usage, however, the closely related term ‘moralism’ is generally applied in a pejorative sense, where the application of a ‘moral frame’ to a particular activity is considered inappropriate or overly demanding. To be ‘moralistic’ or a ‘moralist’ in relation to something or someone, is associated with holding a superior attitude, making proclamations about how others ought to behave, and generally seeking to spoil people’s fun. Moralism has received relatively little attention from philosophers and ethicists, as pointed out (and to some extent, rectified) in a special issue of the *Journal of Applied Philosophy* which took moralism as its focus and provides a very helpful starting point for discussions of this concept (Coady 2005). In her contribution to the special issue, Julia Driver defines moralism as “the illicit introduction of moral considerations” (Driver 2005). Driver identifies the moralist as making a “mistake in judgment and behaviour”. The mistake is generally taken as one of being excessively concerned with morality in some way, and behaving in an inappropriate (didactic) way as a result. Most of the other contributors to the special issue identify moralism along broadly similar lines, as a vice or criticisable in some way, and I follow their lead in this respect.

A difference between moralisation and moralism is that moralism may occur in relation to something within a domain that is (generally) already considered morally-relevant (e.g. one might moralise about someone’s recreational drug-taking). This differs from moralisation, insofar as the latter involves the transition of something from the non-moral to the moral. Moralism is not merely a practice that results in an individual, group, or activity becoming morally-relevant; it is the application of *contentious* moral standards by some people upon the activities of other people. This might be because the standards themselves are contentious, or their particular application somehow raises controversy.

Another distinction between moralisation and moralism is that moralism is something that a particular agent or group of agents *does*, either to another agent or agents, or directed towards trends in society more generally. So, a moralist might lecture a particular drug user on her bad behaviour, or rail against the degradation of society through increased drug use. Moralisation, on the other hand, will not typically have an identifiable individual or group acting intentionally (with regard to the expansion of the moral domain) at its source. Rather, it is the result of a complex interplay of different factors in society. In this way, moralisation refers to a process, whereas moralism to a practice. The terms moralise(d) and moralising can be used for either the process of moralisation or the practice of moralism (e.g. ‘the trend towards teetotalism had a moralising effect on attitudes towards alcohol consumption’; ‘the teetotaller was moralising about her friend’s alcohol consumption’).

These descriptions of moralisation and moralism are here only roughly and imperfectly defined, and I readily accept there will be variation in how others seek to apply these concepts. In particular, disagreement is likely to arise in terms of the contentiousness or otherwise of the application of moral standards. Some will think that one ought to remain neutral as to the appropriateness of expressing moral judgements about people’s behaviour; others will want to capture the negative connotations normally associated with the common usage of these terms. I have sought a compromise here by allowing that paradigmatic moralism (moralist, moralistic) should be disagreeable in some way, whilst moralisation need not be. I do not believe nor claim that there are sharp distinctions to be drawn between the process of moralisation on the one hand, and the practice of moralism on the other, and there will be many examples of overlap. I hope only to provide a rough and ready definition to allow discussion of how moralisation is at work within health promotion.

## Moralising, Health and Responsibility

My concern, in this paper, is with the way in which public health promotion may directly or indirectly lead to the moralising (in both senses) of people’s lifestyles. Health and disease have an interesting relationship with morality. In one sense, they have historically been highly moralised, particularly through religious traditions which link sin and disease, virtue and health. Keith Thomas describes how, in medieval England:

Each of the church’s Seven Deadly Sins was conventionally associated in homiletic literature with a pathological condition of the body… Pride, which was a swelling up, was symbolised by tumours and inflammation. Sloth led to dead flesh and palsy. Gluttony meant dropsy and a large belly. Lust produced fluxes and discharges, leprous skin, and, by the sixteenth century, the pox. Avarice was associated with gout or dropsy; envy with jaundice, venom and fever; wrath with spleen, frenzy and madness.

Thomas 1997: 16-17

Yet in another sense, the medicalisation of conditions is often associated with their removal from the moral domain. For instance, the classification of addictions (to drugs, gambling, and so on) as ‘diseases’ seems to discourage us from making harsh moral judgements about those who are addicted (rightly or wrongly). This may result from enfolding addictions within the biomedical model of disease, one which emphasises the mechanical, de-personalised pathways of ill health and discourages the identification of connections between an individual’s character and her health status, resulting in ‘de-moralisation’ or ‘amoralisation’ (Brandt 1997).

Responsibility seems to play a role in distinguishing cases where health and disease are considered indicative of moral value/disvalue, on the one hand, and those where the recognition of something as falling within the medical domain reduces the extent to which it is subject to moral appraisal, on the other. In the first case, immoral behaviour is thought to result in disease, thus, by observing properly the moral (and, historically, religious) doctrine, one can, to a large extent, avoid disease. In the second, the specification of biological processes which result in what are ordinarily morally criticisable behaviours (such as some anti-social behaviours associated with addiction) distances the agent from those behaviours and emphasises the *lack* of moral responsibility she holds.

Being morally responsible for an action will be a necessary (though not sufficient) condition in order to be morally criticisable for its consequences.^2^ In the case of moralising about health, it is therefore unsurprising that one feature of phenomena that are commonly moralised is that the agent is thought to be morally responsible for them. There is a large philosophical literature that considers the nature and implications of moral responsibility, and how this relates to the legitimate grounds for criticising or praising an agent, which I shall not try to summarise here. However, it is generally accepted that blame, praise, and similar responses should attach only to agents who are appropriately considered morally responsible for their actions.^3^

Two components often taken as required for an agent to be morally responsible are, first, that she had a sufficient degree of control over her behaviour (perhaps fleshed out in terms of her having a *free choice* as to how to behave, or as acting *voluntarily*). And second, that the agent could reasonably be expected to *understand the consequences* of her actions. These are typically referred to as the Control Condition and Epistemic Condition respectively. So, for instance, an agent who has a seizure, during which she injures someone, will not be morally responsible for this violence since she was not in control of her bodily movements at the time. Cases where moral responsibility can be identified and those where an agent is suitably subjected to praise, blame or similar responses are not co-extensive. For instance, an agent who fires a gun, injuring another, whilst under the reasonable belief it was not loaded, may still be considered morally responsible for her actions, at least to some extent, whilst yet being excused from blame or punishment, since she did not foresee the harmful consequences of her behaviour.

There will be other factors important in judging the appropriate application of moral responsibility, such as assessments of the cognitive (and moral) capacity of the agent and how we might use moral responsibility as a way of shaping future behaviour. These will be relevant to the following discussion, but I shall not assess them directly here. Instead, I focus on the importance of perceptions of *control* and *understanding* in relation to the ways in which health promotion can be moralising. I propose that the perceived presence of control over one’s actions, and of a reasonable understanding of the consequences of those actions, will mediate the extent to which agents are deemed to be morally responsible, and consequently, the scope for moralisation.

### Perceived Control and Understanding

I propose that providing information and educating people about the harms of lifestyle-related risk factors encourages the perception that people are both in control of their behaviour, and that they understand the implications of their behaviour for future health. This means that informational / educational approaches to health promotion encourage the view that the two key conditions for moral responsibility are fulfilled. I argue that this plausibly contributes to the view that people are morally responsible for their health-related behaviour and any causally related disease they suffer.

In the context of health promotion, the diagnosis of behavioural risk factors as key to tackling chronic disease, combined with a desire to avoid (actual or perceived) freedom limitation and paternalism, has led to an emphasis on ‘empowering healthy choices’: providing people with the information they need in order to adopt healthy lifestyles. This includes campaigns delivering information about healthy diets, recommended physical activity guidelines, advice on smoking cessation and safe drinking limits, amongst many others. A 2008 King’s Fund report describes how:

In 2005/6 the [UK] government spent more than £30 million on advertising campaigns to stop people smoking, £4.4 million on drug prevention campaigns, and nearly £1 million on the ‘5 a day’ campaign to promote healthy eating… The government also recently announced a £75 million marketing programme to encourage children to exercise and eat healthily

Robertson 2008: 3

The use of education and information provision as a means to change behaviour and promote health indicates that an informational deficit is an important contributing factor to people’s adoption of unhealthy lifestyles, and that rectifying this deficit will help to change those lifestyles. In other words, it assumes that people have unhealthy lifestyles because they don’t know that they’re unhealthy; pointing this out to them will get them to change their behaviour. This is reinforced by the language of ‘empowering’ healthy choices and ‘enabling’ people to make better decisions, often used in relation to these interventions and policies (Woodall et al. 2010; Tones 1991).

The use of the informational deficit model in the context of health-related behaviour has, however, come under increasing criticism from behavioural scientists (Marteau et al. 2008). Evidence suggests that, since many of the behaviours in question are significantly dependent upon environments and engrained habits (and often not under conscious control), factors other than (a lack of) information are likely to be more important factors in determining behaviour (Strack and Deutsch 2004; Marteau 2010; Hollands et al. 2016). Whilst sometimes information provision may be crucial to changing an individual’s behaviour, often people will continue to engage in unhealthy habits despite being aware of the consequences, and often despite expressing a wish to change their behaviour (Sniehotta et al. 2005; Marteau 2010).

Research into the social determinants of health has firmly established that there exists a social gradient in health, whereby those who experience more social deprivation also tend to experience poorer health (including shorter life expectancy and increased rates of disability) (Marmot et al. 2010). It is proposed that structural factors – such as built environment – are important here, along with social and economic differences between those who are more and less socially deprived. For instance, local food environment may influence dietary habits: Macdonald et al. (2007) have shown that deprived areas have a higher density of fast food outlets, plausibly contributing to people’s risk of developing obesity. Such structural factors may mean that, even if educational strategies can successfully raise awareness of behavioural risk factors, people’s capacity to change their behaviour in health-protecting ways may be severely limited by the environments they live in.

Despite this, informational / educational strategies to change behaviour continue to be popular. Kelly and Barker (2016) propose a number of reasons why behaviour change interventions are often poorly designed and likely to be ineffective, many of which relate to the intuitive idea that giving people information and opportunity is likely to lead to healthy behaviour change (Kelly and Barker 2016). I shan’t discuss this here, but one might speculate that it is deeply intuitive as well as reassuring to believe that we are rational agents who respond well to reasons and who are in control of our behaviour: information that contradicts this belief (as evidence on behaviour change seems to) may be resisted.

In summary, much health promotion focuses on achieving behaviour change by providing people with information about the health harms of some behaviours and encouraging them to adopt healthier alternatives. This pushes a message that, through better understanding of the health implications of their behaviour, and given the tools to change that behaviour, people can (and should) adopt healthier lifestyles. This process of creating ‘empowered citizens,’ although often ineffective at actually changing behaviour and improving health, gives the impression that people are, or can become, well-informed and in control of their health. As discussed, this creates the conditions for the attribution of moral responsibility for one’s health.

### Unhealthy Behaviour = Bad Behaviour

Moralisation, as described, is the social process by which activities come to be seen as falling within the the moral domain. Above, we have discussed how health promotion through information provision and education supports the view that people are (or through these forms of intervention, become) in control of their behaviour, and further, sufficiently informed about the impact of their lifestyle to adopt healthier habits. We suggest that this effect, in combination with the trends towards healthism discussed earlier, create the conditions for moralisation by indicating that people have moral responsibility for their health-related behaviour, and that they *ought* to adopt healthy behaviours (given the super-value of health). In this section, we seek to say a little more about how unhealthy behaviour is often viewed as ‘bad’ behaviour, and provide some evidence which indicates that reasoning about moral responsibility (as a product of understanding and control) supports the view that people are appropriately subject to moral criticism for failing to adopt healthy lifestyles.

Public health messages specifying the ‘right’ kind of lifestyle are pervasive, instructing us to (at last count) consume five portions of fruit and vegetables a day, engage in 150 min of moderate physical activity per week, refrain from smoking entirely and drink no more than 14 units of alcohol per week (NHS 2016). The ‘dedifferentiation’ of health – the process of ‘opening up’ health to social, cultural, economic and political interests – and a growing culture of healthism, has encouraged the expansion of health into all areas of life (O’Brien 2003). Since more and better health is always seen as desirable, to fail to pursue this is imprudent, reckless or simply stupid (Bunton et al. 2003).

On top of this, the aesthetic model of health has been perpetuated by commercial interests and media communications (Greenhalgh and Wessely 2004; Ayo 2012). To be healthy is to be beautiful: glowing as you run along the beach at 6 am before returning home to a kale and coconut milk smoothie and 30 min of mindfulness meditation. Through self-improvement in the form of healthy living and self-denial, beauty becomes an attainable goal. The shift from health as freedom from disease to the expansive understanding of health as including complete mental and physical well-being encourages the conflation of all things healthy with all things good and desirable. This aesthetically attractive image of healthiness is accompanied by the symmetrically ugly image of unhealthiness (Gilman 1995). Although physical attraction is clearly deeply context and individual dependent, being overweight or obese is, in much of the world, widely seen as both unattractive and an outwardly visible sign of poor health.

The equation of healthy behaviours with virtuousness, and vice versa, can be more or less explicit. The popular weight loss programme Slimming World uses the metric of ‘syns’ to assist participants with regulating their diet. News and entertainment media are likely to reinforce negative stereotypes of people perceived as having unhealthy lifestyles, particularly those who are overweight or obese (Hilbert and Ried 2009; Puhl and Heuer 2009). The extent and implications of such negative stereotyping, in the case of overweight and obesity, are significant. As summarised by Puhl and Brownell:

Studies on employment have shown hiring prejudice in laboratory studies. Subjects report being less inclined to hire an overweight person than a thin person, even with identical qualifications. Individuals make negative inferences about obese persons in the workplace, feeling that such people are lazy, lack self-discipline, and are less competent… Stigmatization in educational settings seems to take place at all ages. From teasing of obese children to college acceptance, an overweight individual faces serious challenges… One telling study found that parents of overweight children provided them less support for college than parents did for their thin children

The authors go on to propose mechanisms by which such attitudes towards overweight and obese people arise:

Discriminatory attitudes as powerful and consistent as these belie fundamental stigma, bias, and prejudice. These in turn are determined by beliefs that individuals and society have about obese people. These beliefs, it seems, are the confluence of several factors. First, overweight people are assumed to have multiple negative characteristics, ranging from flaws in personal effort (being lazy), to more core matters such as intelligence and being a good or bad person… Second, overweight individuals are believed to be responsible for their condition and that an imperfect body reflects an imperfect person… Finally, whatever bad comes from the bias and discrimination is acceptable, even merited, based on the common belief that people get what they deserve and deserve what they get.

Puhl and Brownell 2001: 801

Further evidence from social science research supports the view that beliefs about controllability, understanding, and responsibility mediate moral ascriptions in relation to people’s health and behaviour. There has been extensive research relating to different aspects of this, that I cannot summarise fully here. However, various studies have shown that, for instance: recognising the causal link lifestyles make to chronic diseases, such as heart disease and cancer, is connected with responsibility and blame ascriptions for those diseases, including amongst physicians (Richards et al. 2003; Bell and Ristovski-Slijepcevic 2015); emphasising the potential for lifestyle modifications to reduce risk of disease affects beliefs about control, blame, and responsibility (Sachs 1996; Brownell 1991; Alicke 2000); health policy that involves coercion or punishment is better accepted where those targeted are considered responsible for the relevant health-related behaviour (Branson et al. 2012); notions of responsibility for disease may moderate the acceptability of using rewards (as well as penalties) as a means of treatment (Promberger et al. 2011); cues about behavioural contributions to disease result in diminished willingness to provide financial support for healthcare costs (Gollust and Lynch 2012).

I have proposed that the presence of control over behaviour and an understanding of the health implications of one’s behaviour, in combination with an acceptance that people ought to pursue healthy lifestyles (and health), indicates moral responsibility for health-related behaviour and subsequent disease. It is one thing to identify what a logical reasoning process would look like, and quite another to show that people *really do* perceive those who suffer chronic diseases as able to control their behaviour / foresee the consequences of their behaviour to an extent that makes them subject to moral criticism, and that those beliefs about control and understanding are affected by informational / educational health promotion. It is, further, an additional step to show that this influence, combined with a culture of healthism, contributes to the moralisation of health-related behaviours. Without claiming to have established any such mechanism, I suggest that a combination of conceptual reasoning and available empirical evidence indicates that such a process is at least plausible, and perhaps a compelling description of the contribution health promotion makes to moralisation. In the final section, I will briefly discuss why moralising health policy should be resisted.

## Against Moralisation in Health Promotion

In this paper, I have sought to outline what moralisation and the related concept of moralism consist in, and to suggest that health promotion can facilitate the ascription of moral responsibility on the basis of health related behaviours, and in so doing, moralise those behaviours and contribute to the moralisation of lifestyles. I have focused particularly on health promotion through information provision and education, since this is a significant area of focus for those trying to effect behaviour change, and an area of health promotion typically seen as benign in comparison to more intrusive interventions such as legal regulation. My concern is that, whilst informational / educational interventions appear to display the virtue of preserving (and, as is often claimed, *empowering*) choice, that very virtue may be the basis for objectionable effects in the form of moralisation.

I suggest that, in general, states ought to avoid enacting policies and practices aimed at health promotion which contribute to moralisation and moralism. First, moralisation (in the sense of social process, as described above) can lead to both direct and indirect harms. Direct harms include those that result from stigmatisation, for instance, as a result of the association of negative stereotypes with particular behaviours and diseases. I highlighted a number of these earlier, as outlined in Puhl and Brownell (2001) in relation to overweight and obesity. Stigmatisation is prominent in relation to other behavioural risk factors for chronic disease, and many of the diseases that result from them: Smoking is heavily stigmatised, as are chronic obstructive pulmonary disorder (COPD) and lung cancer, which are both strongly associated with smoking (Stuber et al. 2008; Chapple et al. 2004; Berger and Kapella 2011). People affected by alcoholism, and resulting liver cirrhosis, also frequently suffer from shame and stigma, meaning they may be reluctant to access treatment services (Vaughn-Sandler et al. 2014; Keyes et al. 2010). Stigma itself is associated with psychological distress, social exclusion, reduced employment opportunities, and other unpleasant effects (Major and O’Brien 2005).

Moralisation may also indirectly harm people, by reinforcing the idea that people *can and should* alter their behaviour. This can lead to overly optimistic assessments of the extent to which information and education are likely to improve health, as discussed in Kelly and Barker’s (2016) analysis of why so many behaviour change interventions are ineffective. Thus, moralisation may contribute to misguided beliefs about appropriate healthcare interventions, and distract from more effective ways of promoting health, leading to opportunity costs. Of course, alternative approaches to health promotion (such as regulation and nudging) may raise ethical concerns of their own. As mentioned, the ethical issues relating to such interventions have already received significant attention in the bioethics literature, and I will not rehearse them here.^4^ If we accept that the state has *either* an obligation to promote health, or that it has an obligation to use resources efficiently (and one of the permissible uses of those resources is to promote health), then the state will also be obliged to adopt methods of promoting health that are effectively, and have good reasons for adopting approaches which are known to be less effective. Such reasons could well be based on ethical considerations (such as avoidance of coercion, distributive justice concerns, and so on). However, these should be made explicit, and the success of an intervention at actually promoting health should be a primary consideration for its introduction.

Much health promotion will stop short of moralism (the practice of subjecting an agent or activity to contentious moral criticism), since it does not include within it any explicit moral criticism of the target individual, group, behaviour or disease. In particular, I do not claim that informational and educational campaigns will generally be moralistic, since they tend not to suggest that those who fail to avoid unhealthy habits commit a moral wrong. Hence, I have focused here on establishing the plausibility of the suggestion that health promotion may contribute to the social process of moralisation, whereby health-related behaviours come to be seen as morally relevant. I do, however, think that moralisation may ‘prepare the way’ for moralism, by increasing the extent to which health-related behaviours come to be seen as morally relevant.

Further, some health promotion *will* be moralistic. So called ‘social marketing’ campaigns make use of stark imagery and simplistic messages – tools borrowed from the sphere of commercial advertising and marketing – in order to highlight the risks of certain behaviours and encourage healthy lifestyles. Images of a foetus formed out of cigarette smoke, or inside an alcohol bottle with the title ‘too young to drink’; a revolver loaded with cigarettes; a young man and woman, apparently drunk, with the message “She’s never cheated on her boyfriend, until now”; a mannequin next to some text saying: “only dummies don’t wear condoms”. Such campaigns appear highly moralistic, equating as they do, specific behaviours (which they frame as health-related) with sinful, bad, undesirable and selfish behaviour. Some commonplace and familiar interventions, such as product labelling on food, cigarettes and alcohol, can also be experienced as moralistic, using disgusting imagery and forcing individuals to be made aware of details about the health harms of the items they purchase, despite some people preferring to remain ignorant of such things (see Bonotti 2014; Loi 2014).

Moralism is objectionable on the grounds that it is overly intrusive and perfectionistic, particularly when deployed by the state. This assumes that states ought to be largely neutral: that they not advantage / disadvantage citizens on the basis of differing reasonable conceptions of the good life (Rawls 1971). Whilst we might accept (albeit with some degree of resentment and frustration) moral criticism by those close to us, the imposition of contentious moral values on us by the state will be unacceptable. Whilst some maintain that a neutrality principle should apply only to coercive state activity, others make a compelling case that state neutrality should extend to all legislation. For instance, Bird (2014) argues that:

[C]itizens in a democratic regime are not bystanders to, but free and equal co-legislators jointly responsible for, public decisions… This lends the need to condone [public policy] crucial salience when some citizens cannot reconcile their conscientious ethical beliefs with the arguments that proponents of controversial legislation make for its enactment.

Bird 2014: 202

If we accept something like Bird’s position that non-coercive state legislation must also be publicly justifiable, according to principles that all reasonable citizens could accept, then health promotion that is directly moralistic, and which facilitates moralism by contributing to the processes of moralisation, should be resisted by the state.

Two likely responses to this are that, first, surely there are some examples of moralisation / moralism, that we would not want to condemn, such as the social change that has made drink driving (in the UK at least) publicly unacceptable and, as a result, has reduced deaths and injuries? Further, and also applicable to the drink driving case, isn’t moralisation / moralism justified in cases where the person *clearly does* commit a moral wrong (such as putting others at significant risk of harm)?

Both these points seem reasonable to me: whilst I have here argued against the use of moralising health promotion, I do not claim that this is inviolable. Where there is compelling evidence to suggest that moralising is a uniquely effective method of reducing harm, then I would likely accept it’s use. Relating to the second argument that moralism just doesn’t always seem objectionable, since it may be correct, in a sense I agree. However, in such cases, the moral criticism does not seem to fall within my description of moralism, since it does not involve the application of contentious moral standards. It is precisely the uncontentious nature of the application of moral standards in cases where there is a clear wrong committed by the agent that makes such cases not examples of moralism, and thus unobjectionable.

## Concluding Remarks

In this paper I have argued that information provision and educational strategies aimed at encouraging healthy behaviour are often taken to be benign. However, I have outlined how such approaches can raise particular ethical concerns. They may do this by contributing to a climate of healthism and reinforcing the moralisation of people’s lifestyles in ways that states ought to resist. I do not argue here that health promotion activities which make use of coercive / non-coercive regulation, or which nudge people towards healthier behaviours are necessarily preferable. Rather, it will be essential to consider any ethical concerns about such interventions in relation, first, to their effectiveness at promoting health, and second, the attractiveness of available alternatives.

## Footnotes

1 For examples of competing accounts, see Boorse 1977; Engelhardt 1976; Ereshefsky 2009; Margolis 1976; Nordenfelt 1995

2 Elsewhere, we have discussed how different varieties of responsibility may be appropriately applied in the context of agents’ responsibility for their health-related behaviours.

3 See, for instance, Strawson’s (1962) seminal essay, which defines moral responsibility precisely as the appropriate invocation of these reactive attitudes.

4 See, for instance, Hausman and Welch 2010; Quigley 2014; Wilkinson 2012; Conly 2012
